# Linking vitamin D levels with recurrent wheeze among Indian children

**DOI:** 10.6026/973206300221289

**Published:** 2026-03-31

**Authors:** Emmadi Vineeth Reddy, Chaparala Sarishma, Bonela Sai Kumar

**Affiliations:** 1 Department of Paediatrics, GITAM Institute of Medical Sciences and Research, GITAM Deemed to be University, Rushikonda, Visakhapatnam, Andhra Pradesh, India

**Keywords:** Vitamin D, atopy, WALRI, rhinitis, wheeze

## Abstract

Vitamin D deficiency (< 20 ng/mL) in children aged 1-5 is linked to higher recurrent wheeze risk via immune dysregulation, but supplementation trials show mixed results. Therefore, it is of interest to assess the association between vitamin D deficiency in children and recurrent wheeze in children aged 1 to 5 years. The total sample size was 80 and the data regarding the patient demographic variables, history of symptoms and allergy, GERD-related recurrent aspiration, history of nebulization, hospital stay etc., were collected using standard Questionnaire. The mean vitamin D level in the study participants was 27.18 ± 7.86 ng/mL and out of 80 cases, the majority, 45% of patients, had WALRI, followed by 27.5% of patients who had pneumonia. Among patients with five wheeze episodes per year, maximum patients 71.4% showed vitamin D levels 11 to 30ng/ml. Thus, Vitamin D supplementation programmes on a national scale help reduce the epidemic of vitamin D insufficiency.

## Background:

In fact, 40% of children will have wheezed at least once by the age of three, and 50% will have wheezed at least once by the age of six [1]. One-third of infants have their first wheezing episode during infancy, and by the time children are six years old, the prevalence of wheeze has increased to about 50% [2]. Oscillations in airway constriction that result in a continuous, melodic sound are the hallmark of wheezing [3]. A critical airway obstruction is more likely to be the cause of wheezing when you exhale [4]. Recurrent wheezers are children who experience three or more episodes of wheezing in a year. Half of the airway resistance in children under five is caused by small-calibre peripheral airways [5]. The relationship between airflow resistance and radius increased to the power of four is inverse. As a result, this child experiences even more acute flow restriction and wheezes when there is even a minor narrowing unlike in adults, wheezing in children can have a variety of causes [6- 7]. The course of treatment is determined by the aetiology, which might be hazardous if improperly followed [8]. Despite India receiving abundant sunlight throughout the year, vitamin D deficiency remains highly prevalent, particularly among otherwise healthy children and adolescents. Several studies conducted across the country have consistently reported low serum levels of 25-hydroxyvitamin D even in children with normal growth and development [9]. This paradox highlights that adequate sunlight availability alone does not guarantee sufficient vitamin D status. Research from India and other parts of the world indicates that approximately 8% to 80% of healthy children suffer from vitamin D insufficiency, which is often associated with inadequate dietary intake of calcium and phosphorus, limited sun exposure, and other lifestyle factors [10]. Because winter sunshine does not encourage the conversion of vitamin D precursors in the skin, those who live at high elevations are more likely to experience seasonal vitamin D shortage. Numerous disorders have been linked to vitamin D insufficiency in recent years, including autoimmune diseases, cardiovascular diseases, malignancies, and type 2 diabetes. Lack of sun exposure, sun-exposed outdoor activities, and inadequate consumption of vitamin D are linked to its inadequacy [8, 10]. One of the most frequent causes of doctor visits and hospital stays for children is respiratory tract infections, which are linked to considerable morbidity and death. Therefore, it is of interest to assess the association between vitamin D deficiency in children and recurrent wheeze in children aged 1 to 5 years.

## Materials and Methods:

## Study site:

The study was conducted in the Department of Paediatrics at a Tertiary care teaching hospital in Visakhapatnam.

## Study population:

Children aged 1 to 5 years with recurrent wheezing.

## Study design:

The current study was an analytical cross-sectional study.

## Sample size:

80 children

## Sampling method:

Consecutive sampling-all patients satisfying the inclusion criteria are included in the study consecutively till the sample size is obtained.

## Study duration:

The data collection for this study took place from September 2022 to January 2024.

## Inclusion criteria:

[1] Children between 1 and 5 years of age who had three or more episodes of wheeze (documented) in the past 12 months were included in the study.

[2] Children whose parents were willing to participate in the study.

## Exclusion criteria:

[1] Children of more than 5 years of age

[2] Children with ongoing vitamin D supplementation

[3] Children with underlying chronic conditions such as foreign body, cystic fibrosis, bronchiectasis, COPD, tumours and structural abnormalities.

## Ethical considerations:

The institutional human ethics committee gave their approval for the study. All participants were asked to sign an informed consent form, and only those who agreed to do so were included in the study. Patients were informed of the potential benefits and risks of the study, as well as the voluntary nature of their participation, before providing consent. The confidentiality of the participants was maintained.

## Data collection tools:

A standardized research proforma was used to record all relevant variables, including socio-demographic information and vitamin D levels.

## Methodology:

Participant's age, sex, the onset of symptoms, and the presence or absence of viral symptoms, including fever, coughing, and dyspnea, were all part of the comprehensive medical histories collected from each patient. We inquired about snoring, noisy breathing, GERD-related recurrent aspiration, and airway abnormalities such as TEF, all of which can be indicators of upper airway narrowing. Based on previous instances of this kind, we wanted to know how serious the problem was; therefore, we asked about the possibility of hospitalization. Questions about the history of nebulization and response to bronchodilators were asked because they help with diagnosis and treatment. People were asked about their own and their family's atopy histories. The history of birth, nursing, child development, and exposure to environmental agents, including pollen, passive smoking, and industrial toxins, was among the topics covered in the questions. A preformatted proforma was used for each of their entries. Vitamin D levels were determined using the CLIA (Chemiluminescent immunoassay) method.

## Statistical analysis:

Once data collection was finished, the information was entered into Microsoft Excel. Data entry errors were rectified after thorough checks of all variables and spreadsheets for completeness. Statistical Package for the Social Sciences (SPSS) for Windows, version 21.0, was subsequently used for data analysis. Tables and figures displaying the results were subsequently labelled with percentages and proportions. Means and standard deviations were used to describe continuous data, whereas proportions and frequencies were used to describe categorical variables. A p-value less than 0.05 were deemed statistically significant for comparing the study variables to generate associations using the chi-square test and the ANOVA test.

## Results:

The majority of patients (27.5%) were 4 years old, followed by 20% of patients who were 3 years old, with a mean age of 3.05 ± 1.37 years (Figure 1 - see PDF). Out of 80 children, 45 (56.2%) were males, whereas 35 (43.8%) were females, indicating a male predominance. Among 80 patients, 44 (55%) had recurrent wheeze for more than 1 year, 32 (40%) had recurrent wheeze for less than 1 year, and 4 (5%) had wheeze since birth. From the results, it was found that the maximum number of patients, 93.7% had cough, followed by 42.5% of patients who had triggers (Figure 2 - see PDF). In the study, 12 (15%) of the patients had rhinitis, and 8 (10%) had eczema. Among the 80 cases, 51 (63.7%) of patients had no family history of wheeze, whereas 29 (36.3%) of patients had a family history of wheeze. In the current study, 21.3% of patients were exposed to passive smoking, 2.5% of patients had exposure to pollen, and one patient had exposure to industrial smoke (Table 1 - see PDF). 52.5% of patients had vitamin D levels of less than 30 ng/mL, with a mean vitamin D level of 20.3 ± 3.32 ng/mL, whereas 47.5% of patients had vitamin D levels of more than 30 ng/mL, with a mean vitamin D level of 34.78 ± 7.86 ng/mL. The mean vitamin D level in the study participants was 27.18 ± 7.86 ng/mL (Table 2 - see PDF). In the present study, among patients with insufficient vitamin D levels, the mean duration of hospital stay was 5.64 ± 1.54 days. In contrast, among patients with sufficient vitamin D levels, the mean duration of hospital stay was 2.58 ± 1.03 days. There was a statistically significant increase in the duration of hospital stay among patients with insufficient vitamin D levels compared to those with sufficient vitamin D levels (P value < 0.0001) (Table 3 - see PDF). Out of 80 cases, the majority, 45% of patients, had WALRI, followed by 27.5% of patients who had pneumonia (Figure 3 - see PDF). In the current study, 40 (50%) of patients experienced three wheeze episodes per year, 24 (32.5%) had four episodes, and 14 (17.5%) had five wheeze episodes per year. Among patients with three episodes of wheeze per year, 17.5% had vitamin D levels of 11 to 30 ng/mL, and 82.5% had levels of more than 31 ng/mL, with a mean vitamin D level of 33.57 ± 4.46 ng/mL. Among patients with four episodes of wheeze per year, 88.5% had vitamin D levels of 11 to 30 ng/mL and 11.5% had levels above 31 ng/mL, with a mean vitamin D level of 22.26 ± 3.83 ng/mL. Among patients with five episodes of wheeze per year, 21.4% of patients had vitamin D levels of less than 10ng/ml, 71.4% of patients had vitamin D levels of 11 to 30ng/ml, and one patient had vitamin D levels of more than 31ng/ml, with mean vitamin D levels of 16.02 ± 5.93ng/ml. There were statistically significantly higher episodes of wheeze among patients with lower levels of vitamin D (P value 0.00) (Table 4 - see PDF).

## Discussion:

Studies show that over 30% of children have at least one wheezing episode before the third year of life, and that incidence rises to over 40% by the time the child is six years old. Recurrent wheeze is a common complaint among paediatric patients [11]. Despite the widespread belief that asthma is the leading cause of wheeze, most children who experience wheezing do not develop asthma as adults. Both during and after pregnancy, the role of vitamin D in the immune system and pulmonary development has been the subject of extensive research [12]. Asthma and other respiratory disorders are more common in people with vitamin D insufficiency, according to multiple research pages [13, 14- 15]. Asthmatic children treated with vitamin D3 experienced fewer exacerbations, required fewer medications, had fewer hospitalizations and showed improved spirometry results, according to recent studies. Increased vitamin D levels or consumption have a protective influence, according to most published studies. However, a few studies also suggest possible detrimental effects [16, 17, 18, 19, 20-21]. In a community-based study that included 901 mother and offspring pairs, researchers found that impaired lung development at age 6, neurocognitive difficulties at age 10, an increased risk of eating disorders in adolescence, and lower peak bone mass at age 20 were all associated with maternal vitamin D deficiency (serum 25-hydroxyvitamin D < 50 nmol/L) at 18 weeks of pregnancy. The results indicate that vitamin D plays a crucial role in fetal development, particularly in the formation of bones, lungs, and brains. Recent studies have shown that the prevalence of this illness can approach 50-90% in India, which was previously considered unusual due to the country's tropical climate. The link between wheeze and vitamin D insufficiency was exaggerated in earlier Indian studies due to the use of antiquated criteria for the illness (< 30 ng/mL). Recent norms in India and elsewhere have redefined vitamin D "deficiency" and "insufficiency" to promote consistency. Low vitamin D levels were the sole recognised reason for frequent wheezing in many children who went to the emergency room for treatment. In the current study, the majority of patients (27.5%) were 4 years old, 20% of patients were 3 years old, 18.7% of patients were 1 year old, 17.5% of patients were 2 years old, and 16.3% of patients were 5 years old, with a mean age of 3.05 ± 1.37 years. A study by Uysalol *et al.*. [22] showed that the mean age of patients with wheeze was 18.05 ± 2.44 months. Whereas Osman *et al.*. [23] reported a mean age of 3.20 ± 0.84 years for patients with wheeze. A study by Gupta *et al.*. [24] showed that 44.2% of patients with wheeze belong to the age group of 6 to 24 months, 29.1% belong to the 25 to 43-month age group, and 26.7% belong to the 44 to 60-month age group. In the present study, 56.2% of patients were males, whereas 43.8% were females, indicating a male predominance, which is in accordance with the results of Uysalol *et al.*. [22] Osman *et al.*. [23] and Salman *et al.*. [24] as well as Samaddar *et al.*. [25] and Gupta *et al.*. [26]. In the current study, 93.7% of patients experienced cough, 42.5% had triggers, 35% had fever and virus-induced symptoms, 21.3% had noisy breathing, and 10% had snoring. Fifteen per cent of patients had rhinitis, and 10% had eczema, which was like the findings of Salman *et al.*. [24]. In the current study, 63.7% of patients had no family history of wheeze, whereas 36.3% of patients had a family history of wheeze. A study by Salman *et al.*. [24] showed that 42.4% of children had an atopic history, 48.5% had rhinitis, 39.4% had allergic conjunctivitis, and 69.7% had asthma in their family. In the current study, 52.5% of patients had vitamin D levels of less than 30ng/ml, with a mean vitamin D level of 20.3 ± 3.32ng/ml, whereas 47.5% of patients had vitamin D levels of more than 30ng/ml, with a mean vitamin D level of 44.63 ± 7.14ng/ml. The mean vitamin D level in the study participants was 31.86 ± 13.38 ng/mL. Uysal *et al.*. [22] reported a mean Vitamin D level of 37 ± 10.1 ng/mL. In contrast, Prasad *et al.*. [27] reported that 23% of children had a very severe vitamin D deficiency, 37.7% had moderate deficiency, 13.1% had deficiency, 26.2% had insufficiency, and no patients had normal levels of vitamin D. The mean vitamin D levels were 11.6 ± 7.4 ng/mL. In the current study, among patients with insufficient vitamin D levels, the mean duration of hospital stay was 5.64 ± 1.54 days. In contrast, among patients with sufficient vitamin D levels, the mean duration of hospital stay was 2.58 ± 1.03 days. There was a statistically significant increase in the duration of hospital stay among patients with insufficient vitamin D levels compared to those with sufficient vitamin D levels (P value < 0.0001). A study by Osman *et al.*. [23] reported mean hospital stay duration of 2.91 ± 0.43 days. Numerous studies conducted on adult and pediatric populations have not demonstrated a discernible improvement in respiratory outcomes, such as upper respiratory infections or asthma, when vitamin D supplementation is taken [28, 29]. Additionally, vitamin D did not affect the length of respiratory support in a trial including extremely preterm newborns [30]. Nonetheless, several observational studies have linked low vitamin D levels to an increased risk of respiratory illnesses [31]. Variations in cord 25(OH)D levels were linked to significant variations in the incidence of respiratory syncytial virus-induced lower respiratory tract infections in term newborns. According to a new meta-analysis of individual patient data, supplementation reduces the risk of respiratory infections, especially in patients who are vitamin D deficient or who take their medication regularly. However, when it comes to the effects of vitamin D on immunological and pulmonary systems, findings from research in other groups shouldn't be applied to premature children who might be in a crucial developmental stage [32]. Numerous studies have found a link between pregnant women who consume less vitamin D and a higher risk of wheezing in their offspring. A recent study indicated that while there was no correlation between breathlessness at one year or four years of age and asthma at age four to six years, superior maternal circulating 25 (OH) absorptions during pregnancy were individually linked to reduced risk of lower respiratory infections in offspring in the first year of life. Although the authors are unaware of the mother's vitamin D levels, they advise that the vitamin D levels of newborns admitted to hospitals due to severe illnesses and persistent wheezing be closely monitored. According to recent research, vitamin D plays a crucial physiological role in both adaptive immunity and innate immunity [33, 34, 35-36].

## Conclusion:

Vitamin D deficiency is significantly more common among Indian children with recurrent wheezing, according to our findings. Vitamin D levels averaged 31.86 ± 13.38ng/ml in patients with recurrent wheezer. Vitamin D supplementation programmes on a national scale could help reduce the epidemic of vitamin D insufficiency. The duration of hospital stay was significantly longer for patients with inadequate vitamin D levels compared to those with adequate levels.

## Declaration of Helsinki:

The author declared that the study was not involved with human subjects or animals subjects.

## References

[1] Weiss LN (2008). Am Fam Physician..

[2] Potter PC (2010). Allergy Asthma Immunol Res..

[3] Kliegman RM (2007). Nelson Textbook of Pediatrics E-Book.

[4] Masoli M (2004). Allergy..

[5] Alonso MA (2019). Pediatr Res..

[6] Smith FM, Saglani S (2023). Expert Rev Respir Med..

[7] Castro-Rodriguez JA (2000). Am J Respir Crit Care..

[8] Al-Shamrani A (2019). Int J Pediatr Adolesc Med..

[9] Boogaard R (2005). Chest..

[10] Carden KA (2005). Chest..

[11] Martinez FD (1995). N Engl J Med..

[12] Brand PLP (2008). Eur Respirn J..

[13] Brehm JM (2010). J Allergy Clin Immunol..

[14] Van Etten (2007). Eur J Immunol..

[15] Zosky GR (2011). Am J Respir Crit Care Med..

[16] Majak P (2011). J Allergy Clin Immunol..

[17] Urashima M (2010). Am J Clin Nutr..

[18] Rajanandh MG (2015). J Pharmacol Pharmacother..

[19] Yadav M, Mittal K (2014). Indian J Pediatr..

[20] Hypponen E (2001). Lancet..

[21] Gale CR (2008). Eur J Clin Nutr..

[22] Uysalol M (2014). BMC Pediatr..

[23] Osman NS (2019). Egypt J Pediatr Allergy Immunol..

[24] Salman MS, Jumah ZM (2020). International Journal of eneral Medicine and Pharmacy (IJGMP)..

[25] Samaddar S (2021). SN Comprehensive medicine..

[26] Gupta P (2016). Indian Pediatr..

[27] Prasad S (2016). J Clin Diagn Res..

[28] Hughes DA, Norton R (2009). Clin Exp Immunol..

[29] Randev S (2018). Indian J Pediatr..

[30] Fort P (2016). J Pediatr..

[31] Alyasin S (2011). Allergy Asthma Immunol Res..

[32] Kloepfer KM (2025). J Allergy Clin Immunol Pract..

[33] Belderbos ME (2011). Pediatrics..

[34] Sharma P (2020). Journal of Pediatric Critical Care..

[35] Giannini S (2022). Nutrients..

[36] Wu Z (2022). Journal of Inflammation Research.

